# *β*-Sitosterol Suppresses LPS-Induced Cytokine Production in Human Umbilical Vein Endothelial Cells via MAPKs and NF-*κ*B Signaling Pathway

**DOI:** 10.1155/2023/9241090

**Published:** 2023-01-03

**Authors:** Yiming Bi, Hongfeng Liang, Xin Han, Kongzheng Li, Wei Zhang, Yigui Lai, Qiang Wang, Xuefeng Jiang, Xiaoshan Zhao, Huijie Fan

**Affiliations:** ^1^Department of Traditional Chinese Medicine, People's Hospital of Yangjiang, Yangjiang 529500, China; ^2^Key Laboratory of Ministry of Education for TCM Viscera-State Theory and Applications, Liaoning University of Traditional Chinese Medicine, Shenyang 110032, China; ^3^The Affiliated TCM Hospital of Guangzhou Medical University, Guangzhou 510140, China; ^4^School of Traditional Chinese Medicine, Southern Medical University, Guangzhou 510515, China

## Abstract

Atherosclerosis (AS) is an inflammatory disease, whose occurrence and development mechanism is related to a great number of inflammatory cytokines. *β*-sitosterol (BS), a natural compound extracted from numerous vegetables and plant medicines, has been suggested to improve AS, but the underlying mechanism remains vague. This work focused on investigating how BS affected the lipopolysaccharide (LPS)-treated human umbilical vein endothelial cells (HUVECs) and further exploring the potential targets and mechanisms through network pharmacology (NP) and molecular docking (MD). According to *in vitro* experiments, LPS resulted in an increase in the expression of inflammatory cytokines like tumor necrosis factor-*α* (TNF-*α*), cyclooxygenase-2 (Cox-2), and interleukin-6 (IL-6). Besides, secretion of IL-6, interleukin-1*β* (IL-1*β*), and TNF-*α* also increased in HUVECs, whereas BS decreased the expression and secretion of these cytokines. NP analysis revealed that the improvement effect of BS on AS was the result of its comprehensive actions targeting 99 targets and 42 pathways. In this network, MAPKs signaling pathway was the core pathway, whereas MAPK1, MAPK8, MAPK14, and NFKB1 were the hub targets. MD analysis also successfully validated the interactions between BS and these targets. Moreover, verification test results indicated that BS downregulated the abnormal expression and activation of MAPKs and NF-*κ*B signaling pathways in LPS-treated cells, including p38, JNK, ERK, NF-*κ*B, and I*κ*B-*α* phosphorylation expressions. Furthermore, p65 nuclear translocation was also regulated by BS treatment. In conclusion, the BS-related mechanisms in treating AS are possibly associated with inflammatory response inhibition by regulating MAPKs and NF-*κ*B signaling pathways.

## 1. Introduction

Atherosclerosis (AS) is a special pathological condition involving endothelial dysfunction, inflammatory infiltration, and plaque formation [1]. The chronic build-up of plaques causes stenosis and thrombosis in sick vessels, while the acute rupture of atheromatous plaques leads to vessel occlusion and critical tissue hypoxia. Typically, myocardial infarction (MI) and stroke, the most fatal complications caused by plaque disruption and thrombosis, have become the major causes of cardiovascular mortality in the elderly, worldwide [2, 3]. The pathophysiological mechanisms of AS are complicated, but evidence has indicated that endothelial cells are the major player in the initiation of AS. Atherosclerotic lesions are frequently formed in certain regions with flow perturbation and endothelial dysfunction [4]. The inflammatory responses of endothelium and vascular leakage have provided the areas for the entry of leukocytes and smooth muscle cells, leading to fatty streak development, which is the earliest visible lesion in AS [5]. The anti-inflammatory strategies of the endothelium seem to be the promising treatments to reduce the risk of atherosclerotic diseases [6]. However, the clinically used therapies for preventing AS are limited to drugs that lower cholesterol levels, while treatments targeting inflammatory properties of AS deserve further investigation.

Phytosterols, a kind of natural dietary micronutrient, are widely distributed in vegetables and fruits such as legumes, seeds, and nuts, which exhibit their potent effects on promoting human wellness in many diseases such as cancers, diabetes, and cardiovascular diseases (CVDs) [7]. *β*-sitosterol (BS), one of the most common components of phytosterols, is the Food and Drug Administration (FDA)-approved potential herbal nutraceutical used to treat AS [8]. Since the chemical structure of BS is similar to cholesterol, the administration of BS can competitively suppress animal cholesterol absorption and reduce serum lipid levels, and it has no deleterious side effects [9, 10]. Some study also indicates that BS improves the viability and morphology of human endothelial cells exposed to oxidized low density lipoprotein (ox-LDL) [11]. In addition, BS inhibits the monocyte chemotactic protein-1 (MCP-1) and intercellular adhesion molecule-1 (ICAM-1) levels in a cell model induced by ox-LDL and TNF-*α*, thus reducing the migration and adhesion of THP-1 monocytes to endothelial cells (ECs) [12]. These results suggest that BS may not only regulate lipid metabolism, but also improve the inflammatory responses of ECs during the development of AS, however, its associated mechanisms remain largely unclear.

With the development of computational methodologies, network pharmacology (NP) has been a novel discipline that can identify drug targets and predict the underlying mechanisms of chemical components [13]. As a branch of NP, molecular docking (MD) can predict the quantitative structure activity relationship (QSAR) between the compound and target, thus achieving the high-throughput screening of herb monomer and providing new insights in the drug discovery and drug design [14]. Recently, these computational methodologies are applied to reveal the regulatory mechanism between the compound and target. For example, Huang et al. successfully predicted and validated the anticancer effects of gentiopicroside by NP [15], and Mu et al. used NP for exploring antiviral actions in rhizoma polygonati [16].

This work focused on investigating if NP and MD were useful for obtaining hub targets, the BS-related molecular mechanisms and its effects against AS. Moreover, this study tried to acquire an objective explanation for validating the prevention of BS in AS by a series of *in vitro* assays.

## 2. Materials and Methods

### 2.1. Materials

BS was obtained in Chengdu Must Bio-Technology (Sichuan, China), whereas LPS (*Escherichia coli*, O55: B5) in Sigma Aldrich (St. Louis, MO, USA). Besides, GAPDH (Cat#3033), p-p65 (Ser536, Cat#3033), p-ERK (Thr202/Tyr204, Cat#4370), p-p38 (Thr180/Tyr182, Cat#4511), p-I*κ*B-*α* (Ser32/36, Cat#9246), p-JNK (Thr183/Tyr185, Cat#58328, Cox-2 (Cat#12282), and GAPDH (Cat#5174) primary antibodies were provided by CST (Beverly, MA, USA). Anti-p65 (Cat#bs-23217R), anti-I*κ*B-*α* (Cat#bs-1287R), anti-p38 (Cat#bs-0637R), anti-JNK (Cat#bs-2592R), anti-ERK (Cat#bsm-52259R), anti-IL-6 (Cat#bs-0782R), and anti-TNF-*α* (Cat#bs-0078R) antibodies were provided by Biosynthesis Biotechnology Co. Ltd. (Beijing, China). Other reagents were obtained commercially.

### 2.2. Cell Culture and Treatment

HUVECs were obtained from Chinese Academy of Sciences Cell Bank (Shanghai, China). After acquisition, cells were cultivated within the endothelial cell medium (ECM), basal medium (Carlsbad, USA) that contained 10% fetal bovine serum (FBS, Gibco, USA) and incubated under 37°C and 5% CO_2_ conditions. This work adopted the thawed cells in the initial 5 passages. Lipopolysaccharide (LPS), an inflammatory agent, was provided by Sigma (St. Louis, USA), followed by dilution to 20 *μ*g/mL with ECM. In the meantime, BS of over 98% purity was obtained in Chengdu Must Bio-technology Co. Ltd (Sichuan, China), followed by dilution to indicated concentrations with ECM before experiments. Then, HUVECs were subject to 24 hours BS pretreatment and later 24 hours LPS treatment.

### 2.3. Western Blot (WB) Analysis

After different treatments, proteins were harvested and their contents were determined by the BCA protein assay kit (Beyotime, China). Thereafter, 10%–12% SDS-PAGE was applied in separating 30 *μ*g proteins, followed by transfer onto the PVDF membranes (Millipore, USA). The membranes were later immersed in 5% BSA for a 2 hours period, followed by overnight incubation under 4°C using the primary antibodies as follows: NF-*κ*B, p-NF-*κ*B, I*κ*B-*α*, p-I*κ*B-*α*, JNK, p-JNK, p38, p-p38, ERK, p-ERK, TNF-*α*, IL-6, Cox-2 (1 : 1000), and GAPDH (1 : 2000). Then, horseradish peroxidase (HRP)-conjugated secondary antibodies (Affinity Biosciences Co. Ltd., China) were added to further incubate membranes. WB assay results were analyzed with the ECL system and ImageJ software.

### 2.4. Enzyme-Linked Immunosorbent Assay (ELISA)

HUVECs were pretreated under different conditions. In line with specific instructions (Dakewe, China), the supernatant was collected and incubated with biotinylated antibody and streptavidin-HRP. The absorbance (OD) values determined at 450 nm were used to calculate the contents of inflammatory cytokines in the supernatant.

### 2.5. Real-Time Quantitative PCR (qRT-PCR)

Inflammatory cytokine mRNA expression was measured by qRT-PCR. TRIzol reagent was added in cells to extract the total RNA, while RNA quality was determined based on the A260/A280 ratio. According to specific protocols, PrimeScript RT reagent kit was utilized to collect cDNA by using gDNA Eraser (Takara, Japan). The SYBR Green (Takara, Japan) was employed for qRT-PCR, while the 2^−ΔΔCt^ approach was utilized to measure gene levels.  Table 1 displays the sequences of all primers.

### 2.6. Immunofluorescence (IF) Analysis

P65, the NF-*κ*B subunit, is related to I*κ*B/NF-*κ*B pathway activation via nuclear import. This work used IF analysis to measure the translocation of p65. In line with specific protocols (Beyotime, China), HUVECs were subject to fixation, washing, blocking, incubation, and staining by diverse reagents. A laser scanning confocal microscope was employed for result observation.

### 2.7. Statistical Analysis

Results were represented by mean ± SD. Statistical analysis was completed with one-way ANOVA, while the least-significant difference (LSD) test was utilized to compare two groups. *p* < 0.05 stood for statistical significance.

### 2.8. Exploration of Potential Mechanisms Based on NP

This work collected the 3D structure format for BS in PubChem (https://pubchem.ncbi.nlm.nih.gov/), and then uploaded it to PharmMapper (https://www.lilab-ecust.cn/pharmmapper/) to screen the putative targets. Other databases, including BATMAN (https://bionet.ncpsb.org/batman-tcm/) and TCMSP (https://tcmspw.com/tcmsp.php) were also utilized to predict the targets of BS. In the meantime, the keyword was “atherosclerosis” for selecting AS-associated genes from OMIM (https://www.omim.org/) and GeneCards (https://www.genecards.org/). Target species of “*Homo sapiens*” was corrected to official symbols by the UniProt database (https://www.uniprot.org/). Moreover, those overlapping targets between BS and AS were acquired using Venn diagrams (https://bioinformatics.psb.ugent.be/webtools/Venn/).

For analyzing target interactions, some bioinformatic methods were applied. This work also built the protein-protein interaction (PPI) network based on different evidence channels via STRING (https://string-db.org/). A high pooled score greater than 0.7 was set and the tsv format file was downloaded. This file was thereafter imported into Cytoscape 3.7.2 for visualization. Gene functions in the PPI network were analyzed through DAVID (https://david.ncifcrf.gov/). With DAVID, Gene Ontology (GO) as well as Kyoto Encyclopedia of Genes and Genomes (KEGG) analysis was conducted for investigating biological progresses together with signaling pathways enriched by BS-related genes against AS.

In addition, the plunge-in CytoNCA of Cytoscape was employed to calculate the “degree centrality (DC)” within genes. The top 30 targets with the highest DC values were selected as the core targets. With the comprehensive analysis of targets, some core targets closely correlated with AS were considered as the hub targets. The 3D PDB files of these hub targets were provided by RCSB PDB (https://www.rcsb.org/search), and then utilized in the MD analysis.

### 2.9. Validation of Hub Genes with MD

In this study, AutoDock Vina was applied to display the binding conformation between BS and hub genes. The crystal structures of hub genes were prepared by AutodockTools to separate local ligand, remove water, add hydrogen, and regulate atom distribution prior to MD, respectively. The 3D structure of BS was also prepared as the ligand. According to the tutorial of AutoDock Vina, the results of semiflexible docking superimposed on the crystal structures of hub genes were obtained. Results with the binding energy <7 kcal were considered as strong combination [17].

## 3. Results

### 3.1. Effects of BS on Inflammatory Factor Levels

**Figure 1:**
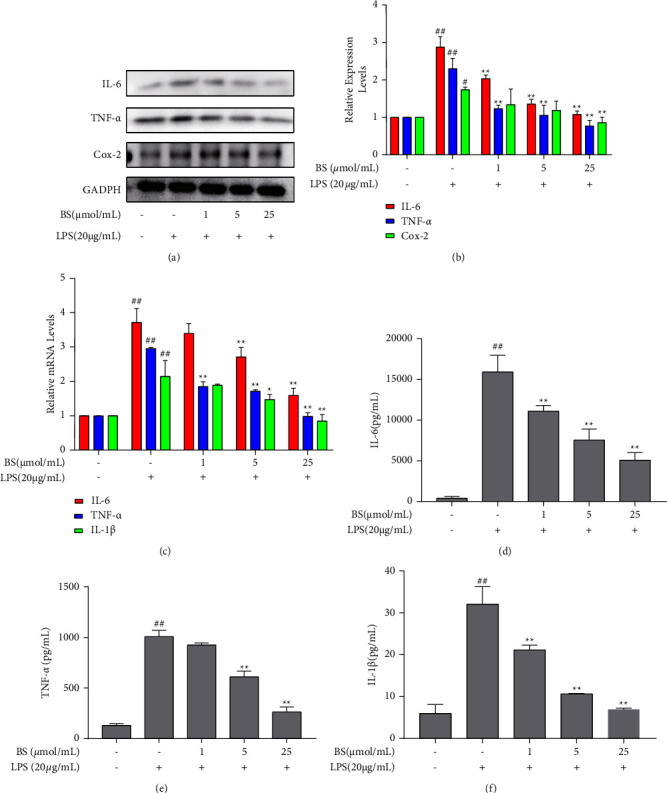
Effects of BS on inflammatory factor levels. (a, b) The expression analysis of IL-6, TNF-*α*, and Cox-2 via western blot. (c) mRNA detection of IL-6, TNF-*α*, and IL-1*β* via qPCR. (d–f) The secretion of IL-6, TNF-*α*, and IL-1*β* via ELISA. ^#^*p* < 0.05 and ^##^*p* < 0.01 compared with the normal control; ^∗^*p* < 0.05 and ^∗∗^*p* < 0.01 compared with the LPS group.

The contents of inflammatory cytokines were measured by WB, qRT-PCR, and ELISA assays. As a result, IL-1*β*, IL-6, TNF-*α*, and Cox-2 levels increased after LPS stimulation. Compared with the LPS group, HUVECs treated with BS produced less inflammatory cytokines, indicating the potential anti-inflammatory effects of BS (Figures 1(a)–1(f)).

### 3.2. Target Identification and Analysis

**Figure 2:**
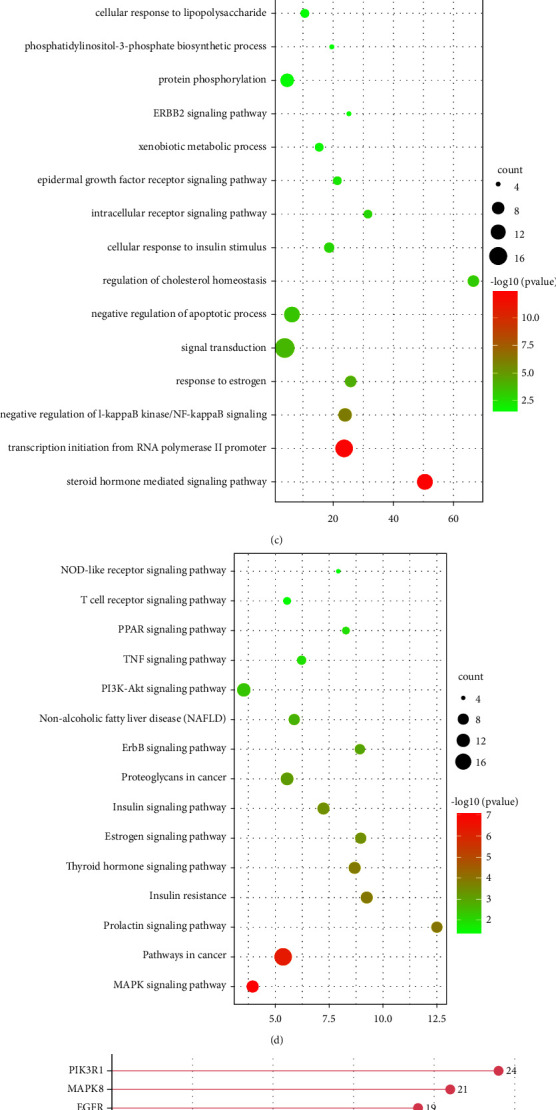
Target identification and analysis. (a) Venn plot of the common targets in AS and BS. (b) PPI network of common targets in AS and BS. (c, d) GO and KEGG analysis of the PPI network. (e) Number of adjacent nodes of key targets between AS and BS.

BS showed its excellent ability to attenuate the expression and secretion of proinflammatory cytokines. In the following analysis, NP was conducted for exploring the BS-related mechanisms against inflammation. This work acquired altogether 139 targets in BS in Pharm Mapper, TCMSP, and BATMAN databases, while 4942 targets related to AS were acquired. After overlapping the targets via Venn diagram, 99 potential targets were finally identified and chosen in the PPI analysis (Figure 2(a)).

The disconnected nodes were hidden, and PPI analysis results are shown in Figure 2(b), with 93 nodes and 314 edges in the network. Nodes were labeled as targets while edges were defined as interactions among nodes. Besides, the font size of the node represented the importance degree in the network. For elucidating BS-related mechanism against AS, GO, and KEGG analyses were performed via DAVID, and results were screened with a false discovery rate (FDR) <0.05. Consequently, this study discovered altogether 43 GO biological processes as well as 42 KEGG pathways. The top five functions were steroid hormone-regulated pathway, I*κ*B kinase/NF-*κ*B pathway negative regulation, transcription initiation from RNA polymerase II promoter, response to estrogen, and signal transduction response. Moreover, KEGG analysis showed that genes were mainly enriched into the cancer pathway, MAPK pathway, prolactin pathway, insulin resistance, and thyroid hormone pathway. According to these results, there were multiple mechanisms of BS against AS, which were mainly correlated with hormone homeostasis, metabolism, and inflammation. The top 15 representative GO terms and KEGG pathways are shown in Figures 2(c) and 2(d). In addition, genes with the highest DC values are listed in Figure 2(e). Some genes such as MAPK8, MAPK1, MAPK14, and NFKB1 were not only the important nodes in the PPI network, but also the major players in biological pathways, which were identified as the hub genes and validated in further study.

### 3.3. Target Validation via MD

**Figure 3:**
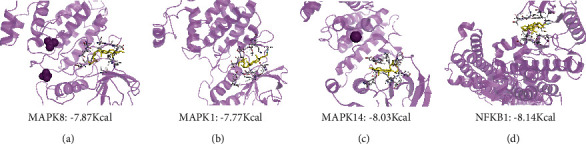
Target validation via MD. (a–d) Interaction analysis between MAPK8, MAPK1, MAPK14, and NFKB1 and BS via molecular docking, respectively.

PDB files of hub genes MAPK8, MAPK1, MAPK14, and NFKB1 (PDB numbers: 2g01, 3w55, 6sfo, and 1nfi, respectively) were downloaded from the RCSB PDB database. MD was performed with the AutoDock Vina software. The best models between BS and targets were identified as those exhibiting the lowest binding energy. The optimal conformations with hydrogen or hydrophobic bonds are presented in Figures 3(a)–3(d). BS contacted with MAPK8 on a stable hydrophobic core consisting of several residues including ASP-169, LEU-168, VAL-40, and ILE-32, and it is also bound to MAPK14 by forming a hydrophobic region around the residues. Besides, hydrogen bonds with GLU-109 (2.81 Å) and MET-108 (2.62 Å) were formed between BS and MAPK1, while a hydrogen bond with GLU-97 (2.31 Å) was also found between BS and NFKB1. The MD results showed the strong binding between BS and hub targets, indicating that the drug-target interactions might be the basis of the biologic activity. This finding also demonstrated that BS exerted its anti-AS effect via the combined action of multiple targets.

### 3.4. Effects of BS in NF-*κ*B and MAPKs Pathway-related Protein Levels

**Figure 4:**
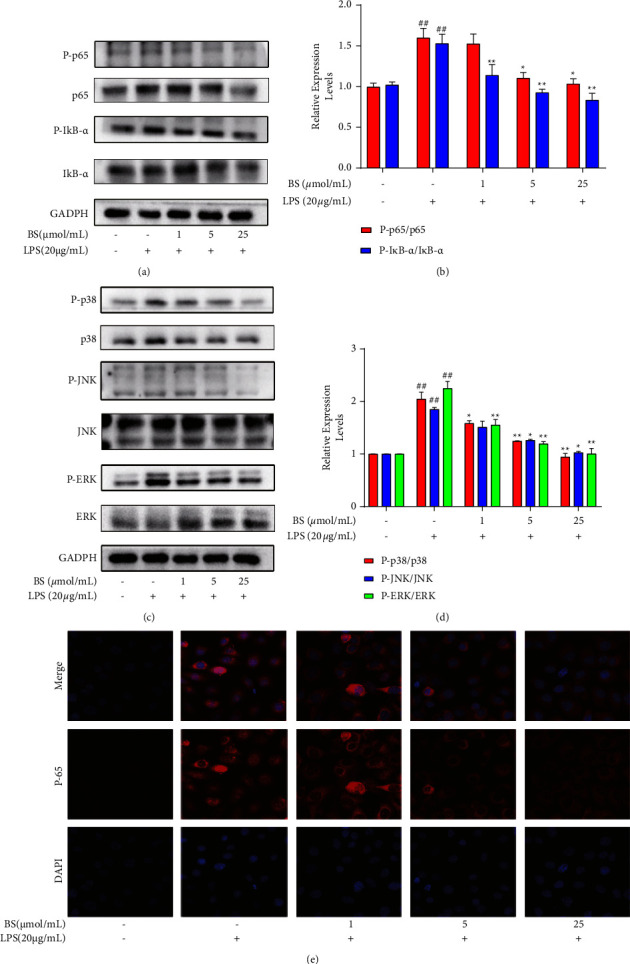
Effects of BS in NF-*κ*B and MAPKs pathway-related protein levels. (a–d) The expression analysis of p-I*κ*B-*α*, p-NF-*κ*B, p-p38, p-JNK, and p-ERK via western blot, respectively. (e) Nuclear translocation detection of NF-*κ*B (p65) from the cytoplasm via immunofluorescence staining. ^##^*p* < 0.01 compared with the normal control; ^∗^*p* < 0.05 and ^∗∗^*p* < 0.01 compared with the LPS group.

As previously reported, MAPK and I*κ*B-*α*/NF-*κ*B pathways have a critical effect on regulating inflammatory cytokine production. After pretreatment with BS (1, 5, and 25 *μ*mol/mL), the phosphorylation levels of I*κ*B-*α* and NF-*κ*B were reduced in HUVECs exposed to LPS (Figures 4(a) and 4(b)). Furthermore, the activation of MAPKs family was enhanced in HUVECs after LPS induction. However, BS declined p-p38, p-ERK, and p-JNK expression within MAPKs family to varying degrees dose-dependently (Figures 4(c) and 4(d)). IF staining revealed the bright red-stained nuclei following LPS treatment, while BS inhibited the abovementioned alterations, indicating that nuclear translocation of NF-*κ*B (p65) was blocked by the BS treatment (Figure 4(e)). All these findings were consistent with their prediction.

## 4. Discussion

Many studies regarding vascular inflammation in atherosclerotic plaques have reported that the imbalance between anti-inflammatory and proinflammatory responses is crucial for AS development. As the most common proinflammatory factors in inflammatory responses, IL-1*β*, IL-6, TNF-*α*, and Cox-2 are recognized to be atherogenic factors. There is evidence supporting that the deletion of TNF-*α* gene reduces the atherosclerotic areas in apoE^−/−^ mice without affecting their serum cholesterol level [18]. Similarly, the plaques narrow in IL-1*β*^−/−^/apoE^−/−^ mice or IL-6^−/−^/apoE^−/−^mice [19, 20]. Some studies have demonstrated that Cox-2 depends on the previous release of IL-1*β* and plays an important role in modulating atherosclerotic plaque stability or instability [21]. Other studies report that these cytokines have a great impact on the vascular homeostasis, particularly in the regulation of vascular permeability, coagulation, leukocyte adhesion, and aggregation, which aggravate the disorders of ECs [22]. Blocking proinflammatory factor levels has been recognized as a candidate approach for preventing AS-related CVDs [6]. Treatments using monoclonal antibodies specifically targeting molecules like IL-1*β*, IL-6, and TNF-*α* markedly decrease the relapsed cardiovascular events among patients developing stable CADs [23].

BS, the dietary phytosterol with bioactivity within plants, is used as a pharmaceutical product for a long history. Though only 5% of BS is obtained from daily intake, it can still lower the serum lipid levels [24, 25]. Besides, BS has displayed its potential *in vivo* and *in vitro*anti-inflammatoryand immunomodulatory activities [8]. To elucidate how BS affected proinflammatory factors, this work measured IL-1*β*, IL-6, TNF-*α*, and Cox-2 levels within LPS-treated ECs; as a result, BS declined these inflammatory cytokines to varying degrees. These results laid the possible pharmacological foundation for the application of BS in treating AS, but detailed mechanisms should be further explored.

With NP, the potential targets of BS to exert anti-AS effects, the biological progresses, signaling pathways, and networks were displayed. A total of 99 targets were screened, which participated in 43 GO biological processes and 42 KEGG pathways. The GO biological processes were roughly divided into three parts, including protein kinase activity, signaling pathway transduction, and hormone regulation. Most biological processes were closely related to the treatment of AS, such as I*κ*B-*α* kinase/NF-*κ*B pathway negative regulation, cholesterol homeostasis regulation, and steroid signaling pathway. Some studies have demonstrated the protective effects of estrogen (a major kind of steroid hormone) in the development of CVDs [26]. High-fat diet can induce atherosclerotic lesion, oxidative stress, ICAM-1, and NF-*κ*B in mice, while these sicknesses of atherogenic diet-fed mice are improved after the estrogen treatment [27]. Our KEGG analysis also showed that the estrogen pathway had a critical effect on treating AS by BS. Other studies have suggested that estrogen receptors (ESRs) encoded by ESR1 and ESR2 may exert favorable effects on cells and molecules implicated in vascular inflammation, which can impede the development of AS [28]. In addition, KEGG analysis also identified other pathways associated with immune and inflammatory responses, such as MAPK, T cell receptor, TNF, and NOD-like receptor pathways. In addition, hub genes were obtained by network analysis. Some targets, such as MAPK8, MAPK1, MAPK14, and NFKB1, were selected as the hub genes for further MD because they were not only the important nodes in the network, but also the major players in the inflammatory signaling pathway. Our MD results showed that BS had a strong binding activity to MAPK8, MAPK1, MAPK14, and NFKB1, respectively. It has been reported that hydrogen bonds and the role of intermolecular forces can be found between small molecule and the active region of target protein. As a result, the small molecule creates inhibition on the target [29], which enables us to conclude that BS can inhibit the activation of MAPK8, MAPK1, MAPK14, and NFKB1. Based on the NP data, it was speculated that the powerful anti-inflammatory effects of BS on AS might be correlated with the inhibition of MAPK8, MAPK1, MAPK14, and NFKB1. NP offers the fast and effective way for drug research; nonetheless, experiments should be conducted to validate targets.

According to our results of *in vitro* study, I*κ*B-*α* and NF-*κ*B activation in HUVECs was remarkably impeded after the BS treatment. Moreover, LPS also activated the subunit of NF-*κ*B (p65), resulting in its translocation from cytoplasm to nucleus. However, BS attenuated this process. The I*κ*B-*α*/NF-*κ*B pathway accounts for a critical pathway that is activated upon inflammatory stress response [6]. The phosphorylation of I*κ*B contributes to NF-*κ*B dimers release from cytoplasmic I*κ*B-NF-*κ*B complex, their subsequent nuclear import, and regulation of genes that encode proinflammatory factors like IL-1*β*, IL-6, and TNF-*α*, as well as the encoding enzymes such as Cox-2 in ECs [30]. Numerous studies have suggested that I*κ*B-*α*/NF-*κ*B pathway suppression can protect ECs from various stimuli in the development of AS. Song et al. reported that I*κ*B overexpression in ECs significantly inhibited the NF-*κ*B activity, mitigated the spontaneous atherosclerotic lesions, and alleviated AS resulting from chronic intermittent hypoxia and cholesterol diet, confirming that the internal endothelial NF-*κ*B pathway was related to atherogenic response [31]. A pharmacologic inhibitor of NF-*κ*B, BAY11-7082 has been proven to suppress proinflammatory factors such as TNF-*α* and IL-6 and inhibit prothrombotic effects for protecting the endothelium [32].

The authors' results also suggested that the activation of JNK, ERK, and p-38 was inhibited in HUVECs exposed to LPS after the BS treatment. In addition to NF-*κ*B pathway, the MAPK pathway is important for regulating cell growth, survival, as well as inflammatory stimulation responses [33]. JNK, ERK, and p-38, which are members of MAPKs family, regulate the proinflammatory genes encoding IL-1, IL-6, TNF-*α*, and Cox-2 during ECs activation and contribute to AS progression [34, 35]. Yamawaki et al. indicated that the normal blood flow restored TNF-*α*-induced inflammation by the inhibition of JNK and p38 MAPK in ECs [36]. Moreover, some studies have suggested that there is cross-talk between MAPKs and NF-*κ*B pathways. Inhibiting MAPKs can inhibit NF-*κ*B phosphorylation, finally blocking the amplification of endothelial inflammation [37], indicating that BS acts via the MAPKs and NF-*κ*B pathways to terminate the inflammatory cascade in ECs.

## 5. Conclusion

By conducting NP and experiments, this study verifies that BS may alleviate the inflammatory cascade via inactivating MAPK and NF-*κ*B pathways within ECs, which may be possible mechanisms of BS in treating AS. However, only *in vitro* assays about inflammation are conducted, some targets related to metabolism and hormone detected in this work must be further explored. Nevertheless, our research not only provides new insights into the pharmacological actions of BS but also lays the scientific foundation for applying BS in treating AS.

## Figures and Tables

**Table 1:** Oligonucleotide and primer sequence.

Names	Primer sequences
GAPDH, human	5ʹ-ATCATCAGCAATGCCTCCTG-3ʹ (forward)
GAPDH, human	5ʹ-ATGGACTGTGGTCATGAGTC-3ʹ (reverse)
IL-6, human	5ʹ-GGTGTTGCCTGCTGCCTTCC-3ʹ (forward)
IL-6, human	5ʹ-GTTCTGAAGAGGTGAGTGGCTGTC-3ʹ (reverse)
TNF-*α*, human	5ʹ-GCTGCACTTTGGAGTGATCG-3ʹ (forward)
TNF-*α*, human	5ʹ-CTTGTCACTCGGGGTTCGAG-3ʹ(reverse)
IL-1*β*, human	5ʹ-AGCTCGCCAGTGAAATGATGG-3ʹ (forward)
IL-1*β*, human	5ʹ-AGTGGTGGTCGGAGATTCGT-3ʹ(reverse)

## Data Availability

The data can be found through the open databases mentioned above.

## References

[1] Gisterå A., Ketelhuth D. F. (2018). Lipid-driven immunometabolic responses in atherosclerosis. *Current Opinion in Lipidology*.

[2] Libby P., Buring J. E., Badimon L. (2019). *Nature Reviews Disease Primers*.

[3] Bentzon J. F., Otsuka F., Virmani R., Falk E. (2014). Mechanisms of plaque formation and rupture. *Circulation Research*.

[4] Gimbrone M. A., García-Cardeña G. (2016). Endothelial cell dysfunction and the pathobiology of atherosclerosis. *Circulation Research*.

[5] Tabas I., García-Cardeña G., Owens G. K. (2015). Recent insights into the cellular biology of atherosclerosis. *Journal of Cell Biology*.

[6] Li B., Li W., Li X., Zhou H. (2017). Inflammation: a novel therapeutic target/direction in atherosclerosis. *Current Pharmaceutical Design*.

[7] Salehi B., Quispe C., Sharifi-Rad J. (2020). Phytosterols: from preclinical evidence to potential clinical applications. *Frontiers in Pharmacology*.

[8] Babu S., Jayaraman S. (2020). An update on *β*-sitosterol: a potential herbal nutraceutical for diabetic management. *Biomedicine & Pharmacotherapy*.

[9] Paniagua-Pérez R., Madrigal-Bujaidar E., Reyes-Cadena S. (2005). Genotoxic and cytotoxic studies of beta-sitosterol and pteropodine in mouse. *Journal of Biomedicine and Biotechnology*.

[10] Yuan C., Zhang X., Long X., Jin J., Jin R. (2019). Effect of *β*-sitosterolself-microemulsion and *β*-sitosterol ester with linoleic acid on lipid-lowering in hyperlipidemic mice. *Lipids in Health and Disease*.

[11] Jiang Y. H., Li X., Niu W., Wang D., Wu B., Yang C. H. (2020). *β*-Sitosterol regulated microRNAs in endothelial cells against an oxidized low-density lipoprotein. *Food & Function*.

[12] Bustos P., Duffau C., Pacheco C., Ulloa N. (2008). *β*-Sitosterol modulation of monocyte–endothelial cell interaction: a comparison to female hormones. *Maturitas*.

[13] Luo T. T., Lu Y., Yan S. K., Xiao X., Rong X. L., Guo J. (2020). Network pharmacology in research of Chinese medicine formula: methodology, application and prospective. *Chinese Journal of Integrative Medicine*.

[14] Ferreira L. G., dos Santos R., Oliva G., Andricopulo A. D. (2015). Molecular docking and structure-based drug design strategies. *Molecules*.

[15] Huang Y., Lin J., Yi W. (2020). <p&gt;Research on the potential mechanism of gentiopicroside against gastric cancer based on network pharmacology</p&gt. *Drug Design, Development and Therapy*.

[16] Mu C., Sheng Y., Wang Q., Amin A., Li X., Xie Y. (2021). Potential compound from herbal food of Rhizoma Polygonati for treatment of COVID-19 analyzed by network pharmacology: viral and cancer signaling mechanisms. *Journal of Functional Foods*.

[17] Li C., Du X., Liu Y. (2020). A systems pharmacology approach for identifying the multiple mechanisms of action for the rougui-fuzi herb pair in the treatment of cardiocerebral vascular diseases. *Evidence-based Complementary and Alternative Medicine*.

[18] Ohta H., Wada H., Niwa T. (2005). Disruption of tumor necrosis factor-alpha gene diminishes the development of atherosclerosis in ApoE-deficient mice. *Atherosclerosis*.

[19] Kirii H., Niwa T., Yamada Y. (2003). Lack of interleukin-1*β* decreases the severity of atherosclerosis in ApoE-deficient mice. *Arteriosclerosis, Thrombosis, and Vascular Biology*.

[20] Elhage R., Clamens S., Besnard S., Mallat Z., Tedgui A., Arnal J. (2001). Involvement of interleukin-6 in atherosclerosis but not in the prevention of fatty streak formation by 17*β*-estradiol in apolipoprotein E-deficient mice. *Atherosclerosis*.

[21] Cipollone F., Fazia M. L. (2006). COX-2 and atherosclerosis. *Journal of Cardiovascular Pharmacology*.

[22] Sena C. M., Pereira A. M., Seiça R. (2013). Endothelial dysfunction - a major mediator of diabetic vascular disease. *Biochimica et Biophysica Acta - Molecular Basis of Disease*.

[23] Ait-Oufella H., Libby P., Tedgui A. (2019). Anticytokine immune therapy and atherothrombotic cardiovascular risk. *Arteriosclerosis, Thrombosis, and Vascular Biology*.

[24] Bin Sayeed M., Karim S., Sharmin T., Morshed M. M. (2016). Critical analysis on characterization, systemic effect, and therapeutic potential of beta-sitosterol: a plant-derived orphan phytosterol. *Medicine (Baltimore)*.

[25] Feng S., Dai Z., Liu A. B. (2018). Intake of stigmasterol and *β*-sitosterol alters lipid metabolism and alleviates NAFLD in mice fed a high-fatwestern-style diet. *Biochimica et Biophysica Acta (BBA) - Molecular and Cell Biology of Lipids*.

[26] Hashemzadeh M., Romo R., Arreguin J. M., Movahed M. R. (2021). The effects of estrogen and hormone replacement therapy on cardiovascular systems. *Future Cardiology*.

[27] Li H., Mani S., Wu L. (2017). The interaction of estrogen and CSE/H(2)S pathway in the development of atherosclerosis. *American Journal of Physiology - Heart and Circulatory Physiology*.

[28] Aryan L., Younessi D., Zargari M. (2020). The role of estrogen receptors in cardiovascular disease. *International Journal of Molecular Sciences*.

[29] Zheng Y., Tian J., Yang W. (2020). Inhibition mechanism of ferulic acid against *α*-amylase and *α*-glucosidase. *Food Chemistry*.

[30] De Martin R., Hoeth M., Hofer-Warbinek R., Schmid J. A. (2000). The transcription factor NF-kappa B and the regulation of vascular cell function. *Arteriosclerosis, Thrombosis, and Vascular Biology*.

[31] Song D., Fang G., Mao S. Z. (2018). Selective inhibition of endothelial NF-*κ*B signaling attenuates chronic intermittent hypoxia-induced atherosclerosis in mice. *Atherosclerosis*.

[32] Vallejo A., Chami B., Dennis J. M. (2018). NF*κ*B inhibition mitigates serum amyloid A-inducedpro-atherogenic responses in endothelial cells and leukocyte adhesion and adverse changes to endothelium function in isolated aorta. *International Journal of Molecular Sciences*.

[33] Cargnello M., Roux P. P. (2011). Activation and function of the MAPKs and their substrates, the MAPK-activated protein kinases. *Microbiology and Molecular Biology Reviews*.

[34] Reustle A., Torzewski M. (2018). Role of p38 MAPK in atherosclerosis and aortic valve sclerosis. *International Journal of Molecular Sciences*.

[35] Zakkar M., D Angelini G., Emanueli C. (2016). Regulation of vascular endothelium inflammatory signalling by shear stress. *Current Vascular Pharmacology*.

[36] Yamawaki H., Lehoux S., Berk B. C. (2003). Chronic physiological shear stress inhibits tumor necrosis factor-induced proinflammatory responses in rabbit aorta perfused ex vivo. *Circulation*.

[37] Yu J., Ming H., Li H. Y. (2019). IMM-H007, a novel small molecule inhibitor for atherosclerosis, represses endothelium inflammation by regulating the activity of NF-*κ*B and JNK/AP1 signaling. *Toxicology and Applied Pharmacology*.

